# A review of neurological health disparities in Peru

**DOI:** 10.3389/fpubh.2023.1210238

**Published:** 2023-09-07

**Authors:** Faris Almubaslat, Sofia S. Sanchez-Boluarte, Monica M. Diaz

**Affiliations:** ^1^Department of Neurology, University of North Carolina at Chapel Hill, Chapel Hill, NC, United States; ^2^Department of Epilepsy, Instituto Nacional de Ciencias Neurológicas, Lima, Peru

**Keywords:** neurological health, disparities, Peru, Latin America, narrative review

## Abstract

Peru is a historically unique and culturally diverse Latin American country. As a low-to-middle-income country (LMIC), Peru faces health implications from the spread of communicable diseases as well as a growing rate of noncommunicable diseases, both of which have been worsened by the recent COVID-19 pandemic’s impact on the national health system. Over the past two decades, the country has aimed to improve health access for its population through various efforts described in this review. Despite this, there are notable neurological health disparities that exist today. This narrative review investigates such disparities through the leading neurological contributors to the national burden of disease in the country, including migraine headaches, cerebrovascular disease, and dementia. Public health disparities that contribute to other major neurological diseases in the country, including epilepsy, neurocysticercosis, Chagas disease, multiple sclerosis, traumatic brain injury, traumatic and non-traumatic spinal cord injuries are also investigated. We also explore potential solutions for overcoming the various neurological health disparities covered in this review that may be applied through public policies, as well as in similar LMICs in Latin America. By overcoming such disparities, the country may be able to successfully address the major contributors of neurological disease burden and create a healthcare environment that can sustainably and equitably improve health outcomes for Peruvian people.

## Introduction

Neurological diseases are major contributors to both disability and mortality worldwide. According to the Global Burden of Diseases, Injuries, and Risk Factors (GBD) study (1), neurological diseases were the leading cause of global disability and the second leading cause of global mortality in 2016 (2). Furthermore, the potential harm from neurological diseases is only increasing. Between 1990 and 2016, the absolute number of deaths and disability-adjusted life-years (DALYs) from all neurological disorders worldwide increased by 39% and 15%, respectively (2). These trends are increasing despite decreases in the number of neurological deaths and DALYs attributed to infectious diseases within that same period (3), highlighting the growing threat of noncommunicable neurological diseases, particularly in low-and-middle-income countries (LMICs).

Factors such as inadequate healthcare coverage and access to resources, growing population size, cultural norms, and unstable political dynamics all detrimentally influence a nation’s ability to manage and overcome the burden of neurological diseases (4). The burden of these diseases is even higher in LMICs (2) that are often less equipped to diagnose, manage, and treat patients with neurological illnesses. For example, LMICs tend to have fewer practicing neurologists and fewer neurology training programs, and clinical guidelines for management of particular neurological conditions often are nonexistent (5, 6). In addition, both diagnostic and therapeutic options related to neurological diseases tend to be disproportionately more expensive and less accessible for people living in LMICs (7, 8). Despite a suspected high burden of both infectious and chronic diseases in many LMICs, the diagnostic imaging and laboratory infrastructure needed to diagnose these conditions is often lacking, causing clinicians to rely on making clinical diagnoses without appropriate diagnostics (9). Infrastructure barriers such as limited public transit options and poor road conditions are often more prevalent in LMICs which can impede access to neurological centers, thereby exacerbating the burden of neurological diseases in affected countries (10, 11).

LMICs are also disproportionately burdened by communicable diseases compared to high-income countries (HICs) (12), and many of those diseases cause neurological symptoms. In addition, the burden of noncommunicable diseases (NCDs) in LMICs has been increasing (3), which is likely a reflection of globalization and its effects on prolonging life expectancy and increasing population growth. The disparity gap between HICs and LMICs has widened further due to the COVID-19 pandemic, increasing the need to better understand neurological disparities in order to appropriately guide public policies and allocate financial resources of the Ministry of Health. For example, LMICs have faced shortages of essential medical resources needed to treat COVID-19 such as ventilators and intensive care unit (ICU) beds that HICs have a greater capacity to access and afford (13). Such shortages have overburdened the healthcare infrastructures of these affected nations, thereby leading to inabilities to adequately treat other diseases, including those with neurological etiologies.

The present narrative review investigates the intra- and inter-country burden of neurological diseases by comparing the current situation of neurological disease diagnosis and care within regions of Peru, an LMIC in Latin America, and also compared with HICs. We explore potential solutions for overcoming these healthcare disparities affecting people living with neurological disease in both Peru and similar LMICs.

## Methods and data synthesis

We performed a comprehensive literature search in Pubmed and Google Scholar up to January 8th, 2023 using the following search terms in combination: “neurological”, “Peru” and “disparities”, as well as each individual neurological condition that is described in the article combined with “Peru” and “disparities”. We also manually searched the reference list of all relevant articles. Both English and Spanish language articles were reviewed (Spanish articles reviewed by authors SS-B and MD). The initial literature search was performed by two independent authors (FA and MD). Duplicate publications were excluded from further evaluation. Given the lack of a standardized definition of “disparity”, the definition that was utilized was the definition recognized by the Centers for Disease Control (CDC): “preventable differences in the burden of disease, injury, violence or opportunities to achieve optimal health that are experienced by socially disadvantaged populations” (14). The neurological conditions discussed in the article were selected by expert opinion (MD and SS-B) or due to a high burden of reported disease in the GBD 2016 study in Latin America (2).

We included peer-reviewed, case–control, cohort studies or clinical trials in humans that took place in Peru, as well as systematic reviews and news press releases on the Peruvian government’s website. Editorials, commentaries, case reports, and preprints were excluded. Screening was performed based on the title or abstract by two reviewers (FA and MD) and disagreements were resolved by consensus or discussion between the two independent authors. To evaluate the relevance and strength of the evidence provided by the studies that were identified, the articles were reviewed by two neurologists (SS-B—a Peruvian neurologist, and MD—a U.S. neurologist with experience working in Peru). Evidence synthesis was based on expert recommendation to include articles from the databases that were felt to be most relevant to the topic. Studies were evaluated based on the reporting of relevant data that would answer the objective question, the validity of the methods used, and the clarity of the results. The authors prioritized research that was considered high quality and insightful to answer the question. In total, 116 full publications were selected to include in this narrative review.

## Demographics and socioeconomics of Peru

Peru is a historically unique and culturally diverse Latin American country with a population of about 33 million people and an estimated annual population growth rate of +1.4% (15). Peru has one of the highest inequality indices in the world highlighting why the healthcare of this socioeconomically and geographically diverse country may serve as a model for analyzing healthcare disparities and inequalities often encountered in similar LMICs (16, 17). Life expectancy in Peru is 77.44 years and the median age is 31.0, both of which are increasing annually (15). However, the proportion of disability-adjusted life years due to NCDs increased from 38.2% in 1990 to 67.9% in 2019 (18). Although factors affecting females such as maternal mortality rates have improved significantly over the past 20 years with a 42% reduction in maternal mortality (19), women often tend to have a higher burden of disease due to lack of access to healthcare (often being the primary caretaker for family members) among other factors (20). Spanish is the primary language in Peru which is spoken by 84% of the population, but regional Indigenous languages, such as Quechua and Aymara, are also spoken throughout the country (21). Ethnically, Peru has a distinctively diverse composition. About 51% of Peruvians self-identify as “Mestizo” (mixed ethno-racial ancestry), while 34% identify as Indigenous Quechua, 5% as Indigenous Aymara, 4% as Indigenous Amazonian ethnic groups, 3% as white, 1% as black, and 2% as other (22). Peru also has a diverse geographic distribution that has gradually been urbanized over time. In 1965, about half the population lived in rural areas, but this has decreased to only about 20% as of 2020, with the remaining 80% now residing in urban settings (23). These ethnic and geographic variations make Peru a country optimal for studying diverse populations, including healthcare disparities encountered between groups (22).

Economically, Peru is considered an LMIC with a gross national income (GNI) *per capita* of $6,030 in 2020 (24). According to the World Bank, this was an 11.3% decline from 2019 (25). Of Peru’s total gross domestic product (GDP), 5.2% was spent on healthcare in 2019 which equates to about $370 per person (25). In comparison to some HICs, the United States spent $10,866 per person on healthcare in that same year, while the United Kingdom, Australia, and Germany spent between $4,500 and $6,500 per person (26). Overall, Peru’s unique demographic composition combined with its limited economic circumstances creates a healthcare environment that is financially restricted and systemically inequitable, which together, give rise to a number of health disparities, particularly for people living with neurological illness.

## Peru’s healthcare system and health barriers

The Peruvian government first set out to provide universal healthcare to its citizens in 1993 following the implementation of several governmental and structural reforms that aimed to improve the country’s overall health and financial well-being (27). With efforts led by the Ministry of Health (MINSA), the country continued its effort to provide universal health coverage and decrease health disparities by restructuring its healthcare system over the following two decades (27). For example, the Comprehensive Health Insurance program, locally known as *Seguro Integral de Salud* (SIS), was established in 2002 which aimed to expand health coverage to citizens of low socioeconomic status (27). Other efforts to improve health access and outcomes included expanding the health workforce by increasing funds to medical schools and nursing programs and requiring newly graduated medical students to work in underserved rural communities through a program called *Servicio Rural Urbano Marginal en Salud* (SERUM) (27). Despite efforts to establish universal health coverage, approximately 13% of Peru’s population remains uninsured as of 2018 (28).

Using the six building blocks for healthcare systems proposed by the World Health Organization (WHO) (29), we describe the various facets of the Peruvian healthcare system to provide a framework for disparities in neurological care in the country:

### Health systems financing

Today, Peru has a decentralized healthcare system that consists of five different entities. MINSA with the SIS program provides health services for 60% of the population, *EsSalud* (Peru’s equivalent of a social security program) provides for 30% of the population, and the Armed Forces (FFAA), National Police (PNP), and the private sector together provide for the remaining 5% (30). The resulting system is made up of multiple providers of both health insurance and health services who often function with a high degree of overlap and little coordination between each other (31).

Listed below are the financing sources of the healthcare sub-systems of Peru:

- Ministry of Health of Peru (MINSA): Tax revenues and user fee co-payments (32, 33).
- EsSalud: A 9% payroll tax paid by the employers of formal-sector workers (33).
- Private Sector: Out-of-pocket expenditures by individual households (33).

### Health service delivery

Despite efforts to expand health access, the Peruvian healthcare system still struggles to provide health-related resources to its rural regions, whose inhabitants are largely Indigenous people that make up approximately 20% of the nation’s population (23, 34). These communities are often located hours from specialty medical centers located in urban cities and many lack the economic and transportation capabilities to travel to these sites. In addition, structural barriers have limited the delivery of medical services and supplies to rural communities (34). Together, these factors have led to long wait times to see providers, contributed to higher rates of infant mortality, and increased levels of malnutrition, infectious diseases, and gastrointestinal problems (35). Overall, geographic barriers to health access, compounded by sociocultural practices that favor alternative methods of medical practice contribute to the health disparities faced by rural and Indigenous communities in Peru. These disparities have led to poorer health outcomes across all fields of medicine, including neurology (36, 37).

### Health workforce

Of Peru’s total healthcare workforce, about 13% are physicians, 13% nurses, and 6% obstetric providers (38). There is an average of 0.82 physicians per 1,000 people (39), less than the United States (2.6 physicians per 1,000 people) (39). As of 2004, there were a total of 254 neurologists working in Peru (40). Today, there are eight hospitals that offer neurology residency training, and two of those also offer pediatric neurology subspecialty training. The majority of Peruvian health professionals are concentrated in urban areas such as the capital city of Lima which has 53 and 40% of the country’s entire quantity of doctors and nurses, respectively (38, 40). In regard to neurologists, this disparate distribution is even more significant. In 2004, 72.8% of all neurologists registered with the Peruvian Medical Association were found to be working in Lima, with another 14.6% working in the second and third-most populated cities of Arequipa and Trujillo (40).

Although there have been efforts to increase medical training, Peru also faces the issue of “brain drain,” the emigration of highly-trained professionals typically from LMICs to HICs, who seek intellectual and financial opportunities outside of their home country. Between 1994 and 2004, Peru lost 13,711 doctors, 7,340 nurses, and 2,112 dentists to outmigration (41). In a 2014 survey of Peruvian doctors, 30% reported intentions of emigrating to another country for better opportunities (36). These significant outmigration patterns impact all specialties of medicine, including neurology, making it crucial to retain neurologists to better address neurological health disparities in Peru.

### Health information systems

Factors including lack of appropriate disease management guidelines and protocols tailored to a country or region and time-efficient disease reporting systems make it difficult for clinicians to obtain current guidelines that reflect the reality where they practice clinically. Lack of internet access and access to computers makes it almost impossible to implement an electronic health record system for accurate patient record-keeping and telehealth services to improve access to care for patients with neurological conditions in rural areas (42).

Peru also faces limitations related to clinical research. A cross-sectional survey of Peruvian neurologists in the major cities of Lima and Arequipa found that the three primary barriers to conducting clinical research were insufficient training in research methodology, inadequate funding, and lack of dedicated time to participate in clinical research (40). Another report found that Peruvian research policies were underdeveloped and that there were limited opportunities to publish in the Spanish language (43). These factors have inhibited the country’s capacity to support clinical investigations into local health issues, including those related to neurological diseases, through standardized research methods. As such, Peru’s underdeveloped research infrastructure creates setbacks in successfully identifying the leading medical disparities facing its citizens and creating solutions to successfully overcome them.

### Access to essential medicines

Access to essential medications for the treatment of neurological diseases is crucial. The WHO Essential Medications List (44) recommends that all countries make medications on the list available *via* public health insurance programs. Despite this, many LMICs continue to face limited medication supplies. For example, new cryptococcal meningitis treatment guidelines published by the WHO recommend a single high dose of liposomal amphotericin B followed by 2 weeks of oral anti-fungal therapy, which can shorten hospital stays and reduce adverse drug effects (45). Unfortunately, in many LMICs, many public sector health facilities do not have access to the liposomal formulation given its high cost (i.e., $2,500–$4,500 US dollars in some private facilities) (46). Inadequate access to healthcare services in rural areas of Peru may also be exacerbated by cultural factors among Indigenous communities where many still seek traditional healers as their primary source of healthcare (35). Traditional healers often perform various spiritual ceremonies and use medicines made from plants, animals, and minerals from the surrounding land to treat disease instead of commercial pharmaceuticals and medical techniques used by healthcare workers (35).

### Leadership and governance

Organized implementation of health policies by governmental entities is a key component to improving healthcare quality and access. Governmental leadership has previously made well-intentioned efforts to improve healthcare for Peruvians such as with the creation of SIS to establish universal healthcare coverage (27). The execution of such efforts has fallen short of effectively providing a quality standard of healthcare, however, especially for lower-income populations (36). Despite the legal availability of universal healthcare coverage that now exists in Peru, between 10% and 20% of Peruvians remain excluded from health services offered by the Peruvian healthcare system (47). Although *EsSalud* has gradually succeeded at increasing health coverage for Peruvians (48), a 2019 study found that in a 3-month period, only 73.1% of program members who sought medical attention actually received healthcare services (48). These deficiencies in access have influenced how Peruvians perceive their government’s ability to provide adequate healthcare availability. A 2022 survey found that approximately 70% of Peruvian adults viewed their access to healthcare as deficient (49).

## COVID-19 pandemic

Toward the beginning of the COVID-19 pandemic, the Peruvian government established a strict nationwide lockdown before the country even recorded its first COVID-related death (50). Despite this, the nation has experienced high transmission and mortality rates from the disease, reaching the highest rates in the world early on during the pandemic (50). These high infection and mortality rates have largely been attributed to the nation’s underfunded health system, its high population density in poor and urban areas, and multigenerational homes (50). In addition, many of Peru’s informal workers, who comprise about 70% of the nation’s workforce, often cannot afford to miss work making it difficult for them to adhere to lockdown restrictions (50). Because Peru’s economy heavily relies on imported goods, Peru has also struggled to maintain sufficient supplies of personal protective equipment, oxygen tanks, and both molecular and antibody tests, further worsening the situation (50). Despite a steady increase in vaccination rates, the outbreak of the Omicron variant led to the highest recorded rates of COVID-19 cases in Peru since the start of the pandemic (51).

In addition to increasing the overall burden of disease, the COVID-19 pandemic has worsened the already growing burden of neurological diseases harming Peru. A recent cross-sectional study of Peruvian patients with mild-to-moderate COVID-19 found that 83% of patients reported at least one neurological symptom (52). Other studies found that stroke caused by COVID-19 infection led to remarkably high rates of mortality among Peruvian patients hospitalized with the disease (53, 54). In addition to the disease having neurological manifestations, the COVID-19 pandemic and the lockdown enacted in response to it both disrupted routine health services in Peru which created barriers to treating many other health conditions, including those of neurological etiologies.

A qualitative study of patients with stroke in Peru found that the pandemic interrupted the continuity of stroke care affecting both patients and their caregivers due to factors such as loss of communication with healthcare personnel and a lack of post-discharge home care training (55). Moreover, inadequate access to rehabilitation services was exacerbated during the pandemic leading to worse functional outcomes (56). Such intersections between COVID-19 and neurological conditions have contributed to the worsening burden of neurological diseases in Peru. Inequalities to healthcare access that have been further exacerbated by the COVID-19 pandemic in Peru include the availability of services and the quality of those services, ethnic and geographic inequities in access to health insurance, and geographic access to healthcare services (57).

## Disparities affecting neurological diseases in Peru

According to the Global Burden of Disease (GBD) study, the total burden of neurological diseases in Peru, as measured in DALYs, notably increased from 9.1 to 10.7% between 1990 and 2015. The adult neurological conditions that contributed most to Peru’s burden of disease in 2015, were migraine, cerebrovascular disease (CVD), and dementia (3). Compared to when data was first collected for the GBD study in 1990, migraine and CVD have remained the first- and second-greatest contributors to adult neurological disease burden in Peru. Dementia, which includes Alzheimer’s and other types of dementia, increased from the fifth- to the third-greatest contributor (3). The next section of this narrative review will examine factors that have exacerbated healthcare disparities of specific neurological conditions with significant morbidity and/or mortality in Peru. Table 1 highlights the disparities encountered for each neurological condition highlighted in this review.

**Table 1:** Within-country and inter-country disparities within a region for each neurological condition covered in this article.

Neurological condition	Contributing health disparities
Migraine	Insufficient access to prophylactic and abortive medications. Depleted medication supply. Expensive user costs. Inadequate health insurance coverage. Stress-induced migraine secondary to poverty-related stressors. Cultural perceptions and expressions of pain. Headache secondary to COVID-19.
Cerebrovascular disease (CVD)	Inadequate access to fibrinolytic therapy. Limited stroke centers, especially in rural regions. Poor traffic conditions. Inequitable access to rehabilitation services. Undereducation about stroke symptoms and the stroke time window. Cultural beliefs about medicine in rural communities. Limited training in primary care for physicians.
Dementia	Health disparities contributing to CVD increasing the development of vascular dementia. COVID-19-related dementia. Increased isolation from the COVID-19 pandemic. Underinsurance among the older adults. Shortage of memory care and mental health facilities. Low number of geriatricians and scarce geriatrics training in medical school curricula. Limited availability to therapeutics. Low awareness and stigma about dementia. Internationally standardized diagnostic tools lacking cultural validity.
Epilepsy	Unavailability of antiseizure medications. Limited access to diagnostics. Seizures related to alcohol use, CNS infections, and traumatic brain injuries.
Neurocysticercosis (NCC)	Suboptimal health environments in rural communities. Underdeveloped water purification and sewage disposal systems. Limited availability of antiparasitic drugs.
Chagas disease	Exposure to livestock reservoirs of *T. cruzi* within rural and suburban communities. Lack of adequate prevention and infection control strategies. Insufficient access to insecticides. Limited effectiveness of trypanocidal medications.
Multiple sclerosis	High cost of disease-specific diagnostics and therapeutics. Low availability of appropriate healthcare resources, especially within rural regions
Traumatic brain injury (TBI)	High frequency of TBI related to road traffic injuries and violence. Limited number of neuro-intensive care units, neurocritical care specialists, and neurosurgeons.
Traumatic spinal cord injuries (TSCI)	Inadequate access to: Acute specialty care services. Ongoing healthcare management. Skilled rehabilitation services. Appropriate assistive devices. Neurosurgeons being limited in frequency and availability.
Nontraumatic spinal cord injuries (NTSCI)	NTSCIs secondary to viral infections with high frequencies in Latin America (HTLV-1, Zika, chikungunya). Undereffective strategies for prevention, diagnosis, and management of such infections

### Migraine headache

From 1990 to 2015, migraine headaches remained the highest contributor to the neurological burden of disease in Peru and increased in terms of DALYs by 57% (3). Specifically, the burden of disease of migraine in Peru in 2015 was 163,433 DALYs, with women more affected than men, and the disease most prevalent among young and middle-aged adults (3). These findings suggest that despite the country’s efforts to improve overall health access and outcomes over recent decades, contributors to the development of migraine remain a significant issue.

One potential issue contributing to migraine prevalence is insufficient access to prophylactic and abortive medications. According to the World Health Organization’s (WHO) Model List of Essential Medicines, it is recommended that every country should, at a minimum, provide access to the following medications that are commonly used to treat migraine headaches: ibuprofen, paracetamol, propranolol, and sumatriptan (44). However, in Peru there are no studies that have analyzed the availability nor the price of these migraine medications. A 2016 study that assessed medication access in Peru found that 30.6% of patients who utilized pharmacies at healthcare facilities for prescription medications experienced insufficient access due to a variety of socioeconomic factors, including poverty and insurance status (58). Moreover, 20.7% reported that they were instructed to fill their prescriptions at an alternative pharmacy (58), suggesting a depleted medication supply. This finding is also reflective of Peru’s fragmented health system that has limited coordination between healthcare sub-systems. Another study found that 47.2% of people who purchased medications in Peru did so without having verified prescriptions from a healthcare provider (59). Qualitative data suggested that this purchasing behavior was largely attributed to delays in medical appointment scheduling and in attending health centers (59), highlighting structural failures related to the provision of healthcare access. Considering these trends related to prescription drug accessibility, it is likely that many Peruvians with migraines are inadequately medicated for the prevention and control of their symptoms.

Socioeconomic stressors are another potential contributor to the persistently elevated burden of migraine that exists in Peru. Several studies have found lower socioeconomic status is a significant risk factor for migraine (60–62) and a contributor to increased frequencies of migraine attacks (62, 63). This association may relate to the levels of stress caused by poverty. There is evidence that suggests repeated or chronic stress can lead to maladaptive changes in neurological pain perception leading to an increased sensitivity and response to pain, known as stress-induced hyperalgesia (64). Patients exposed to high levels of stress from poverty may have an increased risk of experiencing migraines through this process. Food insecurity, lower education, and inadequate access to health services all correlate with lower socioeconomic status and may precipitate stress that can lead to the development of migraine headaches. Furthermore, these factors disproportionately harm rural and Indigenous communities in Peru who are at a greater risk of experiencing poverty and its related downstream stressors. With 20.2% of Peru’s population below the poverty line (65), it is likely that a significant portion of the country’s migraine patients experience such socioeconomic stressors. Moreover, chronic exposure to high altitudes, particularly for those from the Andean region of Peru, is a risk factor for headaches and migraines that may exacerbate its burden in Peru (66). These stressors were likely exacerbated by the COVID-19 pandemic and the resulting lockdown, which led to high levels of job loss and nationwide shortages of imported goods (50).

Cultural perceptions and expressions of pain may be another factor contributing to the apparent burden of migraine headaches on the Peruvian population. According to the GBD study, Peru experiences similar rates of migraine and tension headaches to that of other countries in the northern and western regions of Latin America (67). In comparison, there are notably fewer rates of these diseases in East and Southeast Asia (67). Considering that these regions mostly consist of low- to-middle-income nations and likely have some overlap in socioeconomic stressors, this variation in headache syndrome prevalence may highlight differences in cultural perceptions and expressions of pain.

The COVID-19 pandemic increased rates of migraine more directly as well. In the previously mentioned cross-sectional study that investigated neurological symptoms in mild-to-moderate COVID-19 patients in Peru, headache was the most commonly reported symptom with a response rate of 72% (52). Compared to other studies with similar investigations in different countries, Peruvian patients recorded some of the highest rates of headache (52). This may reflect the fact that the country experienced some of the highest rates of COVID-19 cases throughout the pandemic, in addition to the socioeconomic and system-based disparities previously mentioned.

### Cerebrovascular disease (CVD)

CVD refers to a group of conditions where the flow of blood to the brain is impeded (68). Stroke is one of those conditions, which occurs when an insufficient supply of blood to the brain leads to tissue damage. This damage can cause a variety of neurological symptoms such as weakness, confusion, and vision loss, depending on the location of where the brain damage occurred. CVD has remained the second-greatest contributor to the neurological burden of disease in Peru from 1990 to 2015, even though the DALYs attributed to the disease decreased by 48% (3). This downtrend potentially highlights the country’s efforts to enhance healthcare delivery and health infrastructure during this period, but with CVD remaining a significant disease burden, there is still room for improvement. Although years of life lost (YLL) due to CVD in Peru have decreased since 1990, years lived with disability (YLD) have nearly doubled (3).

Stroke incidence in Peru has been reported as 98.8 per 100,000 person-years with lower incidence among people living in high altitudes (incidence risk ratio 0.09; 95% CI: 0.01–0.63) (69). The age-adjusted incidence of stroke is 93.9 to 109.8 per 100,000 person-years in another study (70), which is high compared with reports from HICs. The burden of CVD in Peru was 149,448 DALYs, with older men aged 60 and above being the most affected population (3). Although length of hospitalization with an acute stroke remained the same between 2002 and 2017 (71), the combined prevalence of ischemic and hemorrhagic strokes in Peru increased by 20.7% (3). In-hospital mortality was 20.8% at post-hospital follow-up with higher mortality in those over age 65 (72).

One potential contributor to the increasing prevalence and persistently high burden of CVD in Peru is inadequate access to fibrinolytic therapy. Despite its known safety and effectiveness for treating ischemic strokes, access to intravenous tissue plasminogen activator (IV-tPA) is a common issue in LMICs (10). In these countries, many of the barriers that prevent the administration of IV-tPA relate to inadequate infrastructure, including a limited supply of the medication, lack of emergency medical transportation services, and inadequate triaging protocols between hospitals (10). One observational study that followed patients receiving IV-tPA at a neurological institute in Peru noted that only 1.98% of stroke patients received IV-tPA due to the majority arriving at hospital centers outside of the 4.5-h window required to administer the medication (10). Common infrastructure-related barriers identified in this study included a lack of comprehensive emergency medical services and poor traffic conditions (10). Especially in rural areas, there remains a lack of readily available stroke centers equipped with the diagnostic tools, medical supplies, and trained personnel required for evaluating and treating suspected stroke patients. In these areas, many hospitals do not have full-time neurologists and most providers lack the training to interpret diagnostic CT scans or to make decisions surrounding IV-tPA administration (10).

Moreover, access to rehabilitation services is limited by financial resources, transportation and delays in referrals. In one study, patients who had a stroke were more likely to have a favorable functional outcome if they were male (relative risk 1.2; 95% CI 1.0–1.5) and patients with public health insurance provided by MINSA were less likely to have a favorable functional outcome (RR 0.7; 95% CI 0.5–1.0) (73). Training of rehabilitation specialists is key to improving the availability of rehab specialists for stroke survivors (74). Educational resources including video tutorials for caregivers of stroke survivors may help improve the quality of care provided by family member caregivers and reduce caregiver burden (75, 76). The barriers to achieving equitable access to stroke care in Peru have likely worsened since the pandemic with COVID-19 cases overwhelming much of the Peruvian hospital system.

Beliefs relating to primary care and prevention are additional barriers that may contribute to the high burden of CVD in Peru. The referenced observational study on IV-tPA administration in the country found that another contributor to delayed administration was a lack of awareness of stroke symptoms or the stroke time window among patients (10). This may reflect a level of limited public health education regarding stroke care amongst the general Peruvian population. The same observational study also noted that many patients with strokes who arrived for care outside of the 4.5-h window were unaware that there were closer healthcare facilities that were equipped to administer IV-tPA and instead traveled a further distance to the more well-known neurological institute (10). As mentioned previously, cultural beliefs surrounding medicine also influence health outcomes for many Indigenous communities in rural Peru who still rely on traditional healers as their preferred option for receiving healthcare. One study found that a community of rural Peruvians preferred to see “*sobadores*,” who are traditional healers who provide massages to treat stroke-affected body parts, before they sought out traditional medical services (77). This was despite already having free access to healthcare through the government-sponsored comprehensive health insurance program (SIS) (77). As for providers, training in primary care is also notably limited within medical training programs in Peru, even though most graduating physicians specialize in the field (36). Only 4–11 of the 298 credits required to graduate from medical school are considered primary care-related (36). These various factors that decrease primary care quality, access to specialists trained in neurological care or stroke care lead to poorer overall health and together contribute to the current disease burden of CVD in Peru.

### Dementia

Alzheimer’s and other dementias (discussed together as “dementia” moving forward) were considered the third-highest neurological burden of disease among Peruvian adults in 2015 (3). Specifically, the burden of dementia was 77,687 DALYs, with women aged 75 and older being the most impacted (3). From 1990 to 2015, dementia in Peru saw an increase in disease burden by 157% (3). This burden increase was similar to that of neighboring Chile, which saw a 200% increase in dementia-related disease burden from 1990 to 2010 (78). Findings from the GBD study indicate that both increases in YLLs and YLDs have contributed to the growing disease burden of dementia in Peru as well as its neighboring countries (3).

A potential explanation for the growing YLD in the region may be that Latin America has seen its life expectancy increase in recent decades, and is projected to continue nearing closer to that of North America and Europe (79). As life expectancy increases, the number of individuals living long enough to develop and live with dementia also rises. As for increases in YLL, this may be attributed to much of the region still being economically disadvantaged, with resulting healthcare access limitations and poor diagnostic capabilities leading to premature mortality (3). A combination of medical, structural, and social factors have likely contributed to the increased disease burden of dementia in Peru, which has a reported prevalence of 6.9% in only one community prevalence study (80). Although the burden of dementia is expected to be elevated in Latin American countries such as Peru, many cognitive screening tests are not validated in populations of lower educational level and low literacy or illiteracy which may lead to under-detection of dementia. It is crucial to validate tests for these populations to accurately understand the true burden of dementia among people of lower literacy or lower educational levels (81).

There are several overlaps between CVD and dementia. Firstly, vascular dementia is a form of CVD that results in cognitive impairment due to multiple infarctions of brain tissue. New studies have also investigated the development of Mixed Vascular-Alzheimer Dementia, which occurs when there is evidence of both diseases present in an individual (82). One study identified a presence of brain lesions suggestive of CVD in Alzheimer’s disease patients, and vice versa with evidence of pathological changes suggestive of Alzheimer’s disease, including amyloid plaques and neurofibrillary tangles, in patients with vascular dementia (83). Considering Peru’s persistently high burden of CVD and the relationship between CVD and dementia, there may be a positive association between the high burden of both diseases in the country.

The relationship between COVID-19 and dementia is another factor that may have increased the burden of dementia in Peru (84). As the pandemic has continued, the scientific community has begun to identify potential links between COVID-19 infection and the development of cognitive impairment. Some of these links include cognitive deficits that persist beyond infection resolution (85, 86) and accelerated development of Alzheimer’s disease pathology and symptoms (87). The COVID-19 pandemic has also created an environment that increased psychosocial risk factors for developing and exacerbating dementia. Particularly in Peru, with its high prevalence of COVID-19 cases and the long-term lockdown that was established in response, the pandemic increased rates of social isolation, loneliness, and depression among older adult Peruvians (88–90). Furthermore, with much of the healthcare system overwhelmed with COVID-19 cases, non-emergent services such as screening and management of dementia have suffered (91).

Beyond the impacts of the COVID-19 pandemic, Peru also faces fundamental structural disparities regarding the delivery of dementia-related services. Peru, like many other LMICs in Latin America, has a shortage of memory care and mental health facilities (92). Healthcare personnel and medical training are also scarce in this field. As of 2014, Peru had only 165 geriatricians to service its older adult population (93) which currently consists of over 4 million people (94). Furthermore, just five of the country’s 34 medical schools had incorporated a geriatrics course into their curriculum (93). In regard to health insurance, even with comprehensive coverage options available such as with SIS, 25% of older adults in Peru remain uninsured, thereby limiting their access to dementia-related healthcare resources (93).

These structural disparities disproportionally harm vulnerable populations. According to the 2016 World Alzheimer’s Report, 83.3% of deaths from dementia occurred in hospital settings among Peru’s rural population, compared to only 29.1% among its urban population (95). This may reflect community differences in the supply of therapeutics, diagnostic tools, and healthcare personnel that can appropriately manage dementia patients. For example, private health insurance companies, which predominantly service 5% of the Peruvian population, are the only providers of acetylcholinesterase inhibitors and glutamate modulators (two medications that have been shown to slow down the progression of memory impairment) in the country (92). Furthermore, most specialists who are trained to diagnose and treat dementia are primarily located in the largest cities of Latin American countries (92).

Such structural barriers are exacerbated by sociocultural factors regarding how dementia is discussed and understood. Both low awareness and stigma around the disease persist throughout Latin America (96). Many older adults in the region remain hesitant about discussing dementia (97, 98), and such feelings often deter people from seeking medical assistance for symptoms of cognitive impairment (95). Stigmatization around dementia in Latin America is especially prevalent in populations with lower education (96). A study in Lima, Peru found that dementia was four times more prevalent among participants who were illiterate compared to those with more than 8 years of education (80). Risk factors for cognitive decline include type 2 diabetes and hypertension in Peruvians older than age 50 in a rural area of northern Peru (99).

Indigenous communities in Latin American countries like Peru are another population found to have higher rates of dementia (97). Standardized cognitive assessment tools mostly originate from high-income Western countries, and they have the potential to lack cultural validity if the word choice and perspective in which certain questions are asked fail to crossover to less similar populations, such as Indigenous communities in Peru (97). This typically occurs when such tools are roughly translated, rather than carefully developed from within target communities (97). For example, if a cognitive assessment tool requires a select list of words to be memorized, that tool may lose its diagnostic accuracy if those words are generally uncommon within the community being evaluated. Moreover, the Peruvian Ministry of Health recommends using the Mini-Mental State Exam which is known to be poorly sensitive for the detection of dementia (100). One study in Peru examined the performance of three commonly used cognitive screening tests and showed major differences in the results of each test highlighting the need for regionally appropriate tools (101). A multination systematic review of Indigenous communities found this to be the primary challenge for diagnosing dementia in this population (102). Such sociocultural barriers in combination with medical and economic disparities collectively contribute to the high burden of dementia experienced in Peru.

## Other neurological diseases in Peru

### Epilepsy

The public health disparities that contribute to the leading neurological diseases responsible for disease burden in Peru extend to other neurological diseases as well. Epilepsy is a brain disorder that causes recurring, unprovoked seizures (103). Seizures are caused by abnormal electrical activity within the brain and are often characterized by sudden and temporary lapses in consciousness, uncontrollable muscle twitching, and urinary incontinence. Whether from a severe head injury, ischemic vessel disease, brain tumors, genetic disorders, or some other reason, epilepsy can originate in a number of ways (104).

In previous Peruvian studies that examined seizure etiologies, the most commonly reported causes were suspension of antiseizure drugs (ASDs), alcohol use, central nervous system (CNS) infections, and traumatic brain injuries (105, 106). In those same studies, a significant number of seizures remained without a determined etiology (105, 106), potentially highlighting disparities in access to needed diagnostic tools in the country. Regarding the frequency of epilepsy in Peru, a study that investigated the disease in rural Peruvian communities revealed that 10.8 out of every 1,000 individuals had active epilepsy, meaning these patients’ seizures were not well-controlled (107). This rate is similar to the findings of a systematic review that investigated the regional prevalence of active epilepsy in Latin America, which was reported as 9.06 per 1,000 individuals (108). Compared to HIC regions across the globe, a meta-analysis of active epilepsy rates noted a lower prevalence of 4.9 per 1,000 individuals (109).

Considering these higher rates of epilepsy in Peru, it is worth investigating the public health disparities that may be contributing to the onset of the disease and potentially preventing access to appropriate management options that are needed to control it. For example, in the same study that investigated the prevalence of epilepsy in rural Peruvian communities, it was noted that only 24% of all epilepsy patients were taking ASDs, all of which were at sub-therapeutic doses (107). This likely highlights a disparity in patient education as well as in access to care. In another study that prospectively followed patients that were admitted to a referral center in Lima with generalized convulsive status epilepticus, a life-threatening manifestation of seizures, only 56.6% of ASDs were administered at their recommended doses (105). In that same study, it was also noted that none of the enrolled participants received an initial electroencephalogram (EEG) for diagnostic evaluation of their seizures (105), even though such instruments are typically the standard of care when managing patients with seizures (110). These disparities in both ASD and EEG availability underscore a need to address the health access barriers that many Peruvians face regarding seizure management. Without such access, Peruvians with epilepsy and other seizure disorders are susceptible to experiencing substandard management and harmful secondary complications compared to their counterparts who reside in HICs.

### Neurocysticercosis

Related to epilepsy, neurocysticercosis (NCC) is the leading cause of acquired epilepsy in Latin America (111). It is a parasitic infection that is caused by the ingestion of tapeworm eggs from the species known as *Taenia solium (T. solium)* (112). Once the ingested eggs enter the bloodstream, tapeworm larvae can travel into the brain where they form cysts, causing NCC (112). After acquiring the disease, some of the most common symptoms include seizures, headaches, and dizziness (112), all of which can be significantly debilitating to the infected individual. NCC can also be fatal if the disease progresses to the development of hydrocephalus, encephalitis, or meningitis (113).

NCC is frequently encountered in Peru. One study that investigated epilepsy in the northern region of the country, considered endemic to NCC, found that 30%–50% of epilepsy cases were associated with NCC (114). Another study that directly examined the prevalence of the disease in a rural Peruvian village found that 36.9% of study participants had antibodies against *T. solium*, however, the majority of those cases were asymptomatic and not classified as NCC (115). Of the participants that were seropositive for such antibodies and underwent CT scans, 18.8% had brain calcifications consistent with NCC, suggesting a high prevalence of the disease (115). Compared to a study that examined the seroprevalence of *T. solium* among a rural, predominately Hispanic community in California, United States, only 1.8% of participants had positive antibodies against *T. solium* (116).

Humans acquire cysticercosis through fecal-oral transmission of *T. solium* eggs by ingesting contaminated sources of water, which are intermediate hosts of the tapeworm (117). In the rural regions of Peru, higher rates of NCC can be attributed to the suboptimal public health environment that places inhabitants at a higher susceptibility to become infected. For example, in such rural communities, pigs often roam freely during daylight hours and are exposed to areas where humans defecate, increasing the pigs’ risk of contamination with *T. solium* which may then be consumed by humans (114). Along with poor hygienic practices, rural Peruvian communities also have less developed water purification and sewage disposal systems, further increasing the risk of human exposure to the parasite (114).

Antiparasitic drugs such as praziquantel, niclosamide, and albendazole may be used for the treatment of *Taenia* species infections (known as taeniasis) (118), however their availability in Peru is limited (111). Considering this limitation, some preventative approaches that can be implemented to control the spread of cysticercosis in LMICs such as Peru include regulating domestic pig-raising practices, tightening slaughterhouse inspection standards, and improving water sanitation and disposal systems (111). Moreover, spreading awareness about hand hygiene practices and providing health education about the risk factors related to the transmission of *T. solium* are likely to reduce the rates of NCC in Peruvian communities (111). Although we know the strategy that can be implemented to eradicate NCC from Peru, public policies must be in place to improve the widespread implementation of these strategies in areas that are highly endemic for NCC in Peru (119).

### Chagas disease

Chagas is another infectious disease that has neurological manifestations and has been exacerbated by the public health disparities in Peru. Also known as American trypanosomiasis, the disease is caused by the protozoan parasite *Trypanosoma cruzi* (*T. cruzi*) (120). The disease is considered endemic to 21 Latin American countries, including Peru, where it has been predominantly transmitted to humans by triatomine bugs, also known as kissing bugs (120). After the acute phase of the disease, which is commonly asymptomatic or presents with nonspecific findings such as a skin lesion, fever, conjunctivitis, and eyelid or facial edema, about 10%–30% of untreated cases progress to a chronic phase with cardiac, gastrointestinal, or neurological manifestations (120, 121). According to a systematic review on the neurological manifestations of Chagas disease, the most frequent symptoms in the acute phase are confusion, headache, hypertonia, seizures, and meningismus, and most often present in children under 2 years of age (122). In the chronic phase, the most common neurological manifestation is neuritis with resulting sensory impairment and tendon reflex alterations, while there are also isolated reports of central nervous system involvement, including dementia, confusion, chronic encephalopathy, and sensory and motor deficits (122).

The triatomine bug that transmits *T. cruzi* is commonly found in the walls or roofs of houses and other building structures that are located in rural and suburban regions (120). A cross-sectional study that investigated *T. cruzi* infection in people residing in rural communities of Northern Peru reported a frequency of 14.9% (123). Urban areas of Peru are also at risk of Chagas disease when triatomine bugs are introduced through the migration of people from rural areas who frequently travel with livestock, including guineapigs, which are an important reservoir of *T. cruzi* (124, 125). Studies that investigated *T. cruzi* infection in peripherally-located urban neighborhoods near the Peruvian city of Arequipa determined a prevalence of between 0.7% and 12.9% in children (124, 126), while another study that screened for the infection among adults attending a cardiac clinic in the city noted a frequency of 5%–6% (124). In addition to the typical route of transmission where triatomine bugs bite the exposed skin of humans and introduce their feces and urine to the bite, other routes of transmission include the consumption of food or beverages contaminated with *T. cruzi*, perinatal transmission from an infected mother to their baby, and transmission from infected blood products or transplanted organs (120).

Containment of this infection is challenging as there is no vaccine currently available for preventing host infection (120). Furthermore, the various routes by which *T. cruzi* may be transmitted more frequently arise in the setting of inadequate health systems that lack adequate prevention and infection control strategies (120). For example, in the study that investigated *T. cruzi* prevalence in rural communities of Northern Peru, it was reported that one community never received household insecticide applications, which is a common method for controlling triatomine bug populations and preventing the transmission of *T. cruzi* (123). Nifurtimox and benznidazole are the only approved drugs that have trypanocidal activity and both are included on the WHO Model List of Essential Medicines (44), making them available for free for Peruvians enrolled in the SIS program. Despite the seeming affordability of these medications, both have been found to have limited effectiveness against chronic forms of Chagas disease (127). They also have severe dose-dependent side-effects such as peripheral neuropathy which occurs in more than 25% of patients who take these medications (127). When considering methods for reducing Chagas disease in Peru, improving access to insecticides in rural communities and investing in research to create safer and more effective trypanocidal medications are both potential solutions. Other proposed methods for reducing *T. cruzi* transmission include providing education on good hygiene practices for preparing, storing, and transporting food, as well as enhancing the screening of donated blood products, organ transplants, and both pregnant mothers and their newborns (120). If Peru were to allocate more resources to the implementation of such solutions, Chagas disease and its neurological manifestations may become less prevalent.

### Multiple sclerosis

Transitioning from the realm of neuroinfectious disease to neuroimmunology, multiple sclerosis (MS) is another neurological disease that is exacerbated by the health disparities in Peru. It is an autoimmune demyelinating disease of the brain and spinal cord that is characterized by the loss of motor and sensory functions, which results in an array of symptoms such as weakness, visual disturbances, abnormal speech, gait and balance difficulties, and bowel and bladder dysfunction (128, 129). The clinical course of MS varies widely, but the disease is typically categorized into two classifications based on the timing of symptoms (129). These classifications are relapsing–remitting MS and progressive MS (129).

Multiple sclerosis affects almost 3 million people worldwide (130), and in Peru, it has been reported to have a prevalence of 9.12 cases per 100,000 inhabitants (131). MS is considered a relatively rare disease compared to other neurological conditions described in this article. The disease is also the leading cause of nontraumatic disability among young adults (132), underscoring the importance to better understanding key stressors of this disease in order to improve outcomes for those who suffer from it, particularly in LMICs.

One key stressor of MS is the cost of management. The expensive costs associated with the diagnostic and therapeutic modalities that are typically used in the standard management of MS place a substantial economic burden on healthcare systems as well as patients, families, and caregivers (129). In the setting of LMICs, where financial flexibility is more limited, these cost barriers are more likely to contribute to rates of underdiagnosis, suboptimal management, and worsened outcomes for patients with MS. For example, a study that investigated access to disease-modifying therapy (DMT) among patients with MS in Latin America, found that only 69.6% of patients in Peru received disease DMT immediately after diagnosis (133). In another study that examined the availability and affordability of frequently used medications (oral prednisone, intravenous methylprednisolone, plasma exchange, intravenous immunoglobulins, azathioprine, rituximab, mycophenolate, and mofetil) in the management of MS and other demyelinating diseases such as neuromyelitis optica and MOG antibody disease across 60 different countries, it was found that these medications were not only less available in LMICs compared to HICs, but fewer than 10% of patients in LMICs could afford such therapies without incurring a catastrophic health expenditure (134). In addition to medications, the use of magnetic resonance imaging (MRI) is key to both the diagnosis of MS and tracking of the disease’s progression in patients (135). This is yet another area where both high costs and low availability contribute to health disparities for Peruvians and other Latin Americans alike who suffer with MS (133, 136, 137). In a similar fashion, oligoclonal band testing through cerebrospinal fluid analysis is another option that can help diagnose MS (part of the revised 2017 McDonald criteria), but is also scarce in availability throughout LMICs (133, 138).

Such barriers to healthcare availability can be overcome, however. In the same study that investigated access to DMT in Latin America, Paraguay, which shares a similar sociocultural and financial profile to Peru (139), achieved access to DMT for 95.8% of people with MS immediately after diagnosis (133). Moving forward, improving MS management in Peru should focus on expanding access to acute management medications and DMTs, as well as to standard diagnostic tools such as MRIs and oligoclonal band testing (133). With most healthcare resources concentrated in urban areas (36), such access barriers will be a greater challenge to overcome in rural regions of the country. The Peruvian Ministry of Health has implemented a public policy (Ley N° 26,842, Ley General de Salud) (140) that has mandated protection and regular vigilance and promotion of rare diseases encountered in Peru. Despite this, lack of imaging and appropriate treatments for MS are lacking in Peru although MINSA continues to make strides to improve healthcare and rehabilitation access for patients with MS, their implementation is limited to large tertiary care referral centers in urban areas (141).

### Traumatic brain injury

Another major cause of neurological disease is physical damage to the brain due to trauma, commonly known as traumatic brain injury (TBI). TBI is a significant cause of death and disability worldwide (142), and typically occurs when the head violently strikes against an object or when an object punctures the skull and damages brain tissue (143). Frequent symptoms associated with TBI include loss of consciousness, headache, dizziness, seizures, confusion, lethargy, vision changes, and alterations in sleep patterns, memory, concentration, and behavior (143). TBI is especially prominent in LMICs, where associated risk factors are more prevalent and restricted healthcare systems limit the management of those harmed by the disease. Specifically looking at Peru, a study that examined the lifetime prevalence of TBI in both rural and urban populations noted a rate of 15.3% and 13.6%, respectively (144).

Based on data from the 1996 GBD study, Latin America and the Caribbean had the highest annual incidence of TBI from road traffic injuries and the second-highest incidence from violence, after Sub-Saharan Africa, compared to all other regions of the world (145). These annual incidence rates were 170.5 and 70.4 per 100,000 inhabitants, respectively (145). This may highlight important areas where public health measures in Peru should focus to lower the rates of TBI in the country. Similar to other Latin American countries, there also remains a need to increase the number of neuro-intensive care units in Peru to improve the management of TBIs and other life-threatening neurological conditions (146). Expanding the number and availability of trained neurologists, including specialized neuro-intensivists, as well as neurosurgeons is also imperative for improving Peru’s approach to TBI management (146).

### Traumatic spinal cord injuries

Moving down from the brain, disorders of the spinal cord should also be considered when reviewing how public health disparities impact neurological disease. Spinal cord disorders are commonly classified as “traumatic” and “nontraumatic.” The symptoms of such disorders, also known as traumatic spinal cord injuries (TSCIs), typically depend on the severity and the location of the injury along the spinal cord. The most common symptoms of TSCIs include pain, weakness, fatigue, and numbness (147), while those with severe forms of injury can also experience symptoms of dysautonomia, such as bowel or bladder dysfunction and changes in heart rate, blood pressure, or breathing (148, 149).

According to the WHO, TSCIs annually harm between 250,000 and 500,000 people around the world (148). In LMICs such as Peru, individuals who suffer from TSCIs face worse survival rates compared to those in HICs (148). The WHO attributes these lower survival rates among victims of TSCIs in LMICs to a combination of health system shortcomings, including inadequate access to acute specialty care services, ongoing healthcare management (such as monitoring for UTIs in incontinent patients), skilled rehabilitation facilities, and appropriate assistive devices (148). The GBD study reports that in 2016, the prevalence of TSCIs in Peru was around 622 per 100,000 inhabitants with an annual incidence of 282 per 100,000, underscoring the notable impact that the disease has on the country’s population (150). Moreover, neurosurgeons are mainly available in private hospitals with few in public hospitals in Peru, therefore, increasing access to neurosurgical services in the country is crucial to improve acute management and long-term outcomes of patients with acute TSCI in Peru.

Global trends depict that the majority of TSCIs with preventable causes are attributed to motor vehicle accidents, falls, or violence (148). These risk factors should be a focus for Peruvian health officials to combat the number of preventable TSCIs in the country. Furthermore, potential solutions are likely to subsequently prevent other neurological diseases caused by physical trauma, including TBIs, which as discussed previously, share many of the same risk factors.

### Nontraumatic spinal cord injuries

Although up to 90% of spinal cord injuries are due to traumatic causes, the proportion of nontraumatic spinal cord injuries (NTSCIs) seems to be increasing (148). In fact, the incidence of NTSCIs has been reported to be higher than that of TSCIs in several countries, with the best evidence supporting these rates noted in studies conducted in Australia and Canada (151–153). NTSCIs have a number of different etiologies, including autoimmune demyelinating diseases, degenerative conditions, genetic disorders, viral and bacterial infections, neoplasms, vascular ischemia, and arteriovenous malformations (151). Like traumatic forms of spinal cord injury, the symptoms of NTSCIs typically depend on the severity and location of the pathology along the spinal cord and are often similar in presentation (154).

Although epidemiological data surrounding NTSCIs in Peru, and in Latin America as a whole, is limited (155), certain etiologies that more frequently occur in the region may expose underlying health disparities that can be addressed to lower the burden of this disease. In one Peruvian study that investigated the disease among hospitalized patients at the National Rehabilitation Institute in the city of Callao, 37.6% of patients with NTSCIs had an infectious etiology (24.3% viral and 13.3% bacterial), with viral transverse myelitis and Human T-cell lymphotropic virus type 1 (HTLV-1) being the most predominant of those infectious etiologies (155). Following infectious causes, degenerative diseases such as spinal stenosis and herniated discs were responsible for 15.7% of NTSCI cases, followed by neoplasms (13.8%), developmental disorders (9.5%), vascular diseases (9%), and idiopathic causes (5.7%) (155).

With viral infectious etiologies being the most common cause of NTSCI in the Callao study (155), and considering that viruses such as HTLV-1, Zika, and chikungunya (all of which have associations with NTSCIs) are disproportionally more prevalent in Latin America compared to most other regions of the world (156–158), public health officials in Peru should aim to reduce the spread of such infections to secondarily reduce the prevalence of NTSCIs. Improving prevention strategies against such viruses and expanding the national health system’s capacity to identify these diseases before they progress to NTSCIs may provide significant relief to the harm caused by this condition.

Overall, the wide variety of neurological diseases that are exacerbated by public health disparities in Peru stresses the importance of finding solutions to such barriers. In turn, effective public health strategies will hopefully reduce the neurological disease burden on the country’s population and reduce the strain caused by these conditions on the nation’s healthcare system.

## Conclusions and moving forward

Peru is a unique LMIC in Latin America with a diverse multiethnic and multilingual population. Despite governmental efforts to improve health coverage beginning 21 years ago, the current Peruvian healthcare system has had shortcomings regarding the provision of comprehensive and equitable care to its population. Specifically, low-income, rural, and Indigenous communities have been some of the most impacted by the public health disparities stemming from such shortcomings. These disparities extend to the burden of neurological diseases in Peru which are most represented by migraine, CVD, and dementia, but also impact a variety of other diseases including epilepsy, neurocysticercosis, Chagas disease, multiple sclerosis, TBI, and both traumatic and nontraumatic spinal injuries. A variety of economic, structural, and sociocultural barriers have contributed to the burden of these neurological diseases, which have only worsened due to the direct and indirect impacts of the COVID-19 pandemic. Moving forward, political, medical, and public health leaders should work together to create potential solutions for overcoming such barriers related to neurological disease.

Accessibility remains a problematic cause of neurological health disparities in the country, and improved efforts to both expand insurance coverage and enhance the availability of medical facilities, diagnostic tools, and therapeutics are key to improving health outcomes. Increasing national health spending to expand the number of adequately equipped medical centers that can evaluate and treat neurological emergencies would help address health system shortages that contribute to the aforementioned disease burdens. Furthermore, improving national road and public transit infrastructures would help address geographic disparities that disproportionately harm rural communities.

There is also a need to increase and retain neurology-trained specialists in the country. This can be addressed by expanding training in the field and creating financial incentives to counter the outmigration of neurologists to HICs (36, 40). Improving Peru’s clinical research capacity would be another incentive that can help retain the national workforce of neurologists. This can be accomplished by expanding research methodology training, increasing grant funding for neurology-related projects, and allocating dedicated time for physicians to participate in clinical research activities (40). Another potential solution for preventing the outmigration of neurologists and improving Peru’s research capacity is to increase collaborative research and training efforts with other nations. Through these proposed efforts, Peruvian physicians would have the opportunity to engage with the health systems of both developed nations and nearby Latin American countries that offer neurological subspecialty education in order to receive training without the need to emigrate abroad (6, 40, 159). Furthermore, such collaborative projects can expand access to funding by receiving support from the multiple institutions that would be involved in these efforts (3). By retaining and expanding the nation’s supply of neurologists, as well as enhancing their ability to partake in clinical research initiatives, the neurological disparities facing Peru could be better investigated and more readily addressed.

Prioritizing preventative medicine and primary care is another route for improving neurological disparities. A potential option to achieve this is to modify the Peruvian medical training curriculum to focus more on neurological care during medical school and residency. Improving incentives for primary care doctors to serve in lower-income and rural communities is another solution that Peruvian health leaders can employ. It is important for primary care doctors to receive education either *via* online webinars or teachings on diagnosis and management of common neurological conditions, so that they can be managed by the primary care provider or an appropriate referral to a neurologist can be placed. For example, management of migraine, stroke, controlled epilepsy or neurocysticercosis could be managed by a primary care doctor if educational curricula were provided and required by the licensing board on neurological conditions. This would help prevent the need for a referral to a neurologist many of whom are concentrated in the capital, Lima.

For both the Peruvian public and the country’s medical workforce, sociocultural stigmas around dementia and primary care remain evident, and there is a need to normalize discussions around these topics, increase education about them, and improve the cultural sensitivity of diagnostic tools used for affected patients. Through the Ministry of Health, the creation of educational public health campaigns could help raise awareness, normalize discussions, and improve understanding of these topics, nationwide.

Finally, as the COVID-19 pandemic persists, there remains a need to both further understand and improve the management of neurological symptoms that stem from COVID-19 infection. It is also imperative to develop solutions to the health access barriers that were exacerbated by the national lockdown, which has restricted needed medical services to Peruvians throughout the country. Increased efforts to improve testing and vaccination would help address these issues by lowering the number of COVID-19 infections and hospitalizations and decreasing the overall stress on the Peruvian healthcare system.

Overcoming the disparities highlighted in this review will be challenging, however comprehensive efforts to address the major contributors to neurological disease burden in the country will hopefully create a healthcare environment that can sustainably and equitably improve health outcomes for the Peruvian people.

## Author contributions

FA: writing, drafting of content of manuscript. SS-B and MD: review of critical content of manuscript. All authors contributed to the article and approved the submitted version.

## Funding

MD is funded by the NIH NIMH (K23MH131466), Alzheimer’s Association (AARGD-22-924896), American Academy of Neurology.

## Conflict of interest

The authors declare that the research was conducted in the absence of any commercial or financial relationships that could be construed as a potential conflict of interest.

## Publisher’s note

All claims expressed in this article are solely those of the authors and do not necessarily represent those of their affiliated organizations, or those of the publisher, the editors and the reviewers. Any product that may be evaluated in this article, or claim that may be made by its manufacturer, is not guaranteed or endorsed by the publisher.
